# Polymers containing phosphorus groups and polyethers: from synthesis to application

**DOI:** 10.1186/1752-153X-6-132

**Published:** 2012-11-08

**Authors:** Smaranda Iliescu, Leire Zubizarreta, Nicoleta Plesu, Lavinia Macarie, Adriana Popa, Gheorghe Ilia

**Affiliations:** 1 Institute of Chemistry, Romanian Academy, 24 Mihai Viteazul Bvd, Timisoara 300223, Romania; 2 Instituto Tecnológico de la Energía, Av. Juan de la Cierva, 24 Parque Tecnológico de Valencia, Paterna, Valencia, 46980, Spain

**Keywords:** Phosphonate-PEG polymers, 1-methylimidazole, Acid scavenger, Solid polymer electrolyte, Limiting oxygen index

## Abstract

**Background:**

Phosphorus-containing high performance polymers have aroused wide interest, mainly due to good mechanical properties and their excellent fire resistance. The flexibility of synthetic polyphosphoesters allows the development of polymers in order to obtain solid polymer electrolytes for rechargeable lithium batteries based on solid films with superior fire resistance.

**Results:**

Novel linear Phosphonate-PEG polymers were synthesized by solution polycondensation of 4-chlorophenyldichlorophosphonate as a linking agent and poly(ethylene glycol)s with different molecular weights in the presence of triethylamine or 1-methylimidazole as acid scavenger. The yields were between 54% and 88% and inherent viscosity between 0.18-0.48 dl/g. Molar masses, Mn were about 26300 g/mol for polyphosphonates with PEG 2000 and 4600 g/mol for polyphosphonates with PEG 200. The LOI values for these polymers and membranes are in the range of 26–29. The membranes based on polyphosphonate with PEG 200 and 2000 showed conductivity between 6 × 10^-8^ S.cm^-1^ and 6 × 10^-7^ S.cm^-1^ at room temperature and total ionic transference number between 0.87- 0.96. The evolution of conductivity vs. temperature is linear.

**Conclusions:**

1-methylimidazole was found to be better HCl scavenger than triethylamine, and allowed higher yields and more eco-friendly synthesis of the Phosphonate-PEG polymers for SPE. These polymers and membranes based on these polymers showed good LOI values and indicate an improvement of the safety of lithium batteries. The membranes present conductivities around 6 × 10^-7^ S.cm^-1^at room temperature and total ionic transference number is higher for membranes based on polymers with high EG unit content. Best results yield 88%, inherent viscosities 0.48 dl/g and Mn 26000 were obtained with 1-methylimidazole and PEG 2000. These membranes based on these polymers showed good LOI values (in the range 26-29%) and indicate an improvement of the safety of lithium batteries.

## Background

Polymers containing phosphorus in the backbone have been intensely studied during the last years. They have aroused wide interest, mainly due to their excellent fire resistance and good mechanical properties [1-3]. Polyphosphoesters can also form another interesting class of phosphorus containing polymers which were developed as biomaterials [4].

The synthetic flexibility of polyphosphoesters allows the development of copolymers with poly(ethylene glycol), poly (ethylene oxide), poly(propylene oxide) etc. in order to obtain solid polymer electrolytes. Interest in the development of rechargeable lithium batteries based on solid polymer films as electrolytes has increased in recent years. Lithium batteries are an important power source for small electronic technologies because of desirable characteristics including high-energy density, low weight, and excellent cycle performance [5,6].

The battery is constructed as: cathode, electrolyte and anode. There are three basic categories of electrolyte materials for lithium batteries: liquid, including organic solvent-based electrolytes; inorganic solvent-based electrolytes; and molten salts (low temperature = ionic liquids); solid: solid polymer, ceramic, gel, and glassy electrolytes; composites [7].

Besides, Solid polymer electrolytes (SPE) are promising materials for electrochemical device applications, namely, high energy density rechargeable batteries, fuel cells, supercapacitors, electrochromic displays etc. [8].

Solid polymer electrolytes must exhibit high ionic conductivity of about 10^-3^ Scm^-1^ at ambient temperature and good mechanical strength. They should also possess wide electrochemical stability, typically between 0–4.5V for single cell and good compatibility with high voltage cathodes such as LiCoO_2_, LiNiO_2_, and low voltage anodes such as lithium, Li-graphite, Li-Sn. For solid polymer electrolytes the main disadvantage, limiting their further development, is related to their very low conductivity at room (ambient) temperature - usually below 10^-5^ S cm^-1^ - and low lithium transference number (usually, in 0.2-0.3 range) [9].

Solid polymer electrolytes have been extensively studied in the past three decades since the pioneering studies of materials based on the complexes of poly(ethylene oxide) (PEO) with alkali metal salts were reported by Peter V.Wright and M.B. Armand [10,11]. They have shown that poly(ethyleneoxide) (PEO) (CH_2_CH_2_O)*n* can act as a host for sodium and potassium salts, thus producing a solid electrical conductor polymer/salt complex.

An important class of solid polymer electrolytes is based on poly(alkyelne glycols). Poly(ethyleneglycol) (PEG) with the chemical formula H(−O––CH_2_–CH_2_)_n_–OH, has the same monomeric unit as PEO but has an end hydroxyl group. Poly(ethylene glycol) has high solvating ability for inorganic salts and shows homogeneous solution mixing because of interactions with polar ether groups and coordination with dissociated cations. Solid polymer electrolytes consisting of PEG of molecular weight 2000 and LiClO_4_ were prepared [12]. (PEG)*x*LiClO_4_ shows ionic conductivity of 7.27 × 10^–7^ S^.^cm^-1^ and a high value of ionic conductivity ~ 10^–4^ S^.^cm^-1^ at a temperature close to the melting point.

Several another polymers have been used for the purpose of designing new solid polymer electrolytes that include poly(siloxanes), poly(phosphazenes), poly(vinylpyrrolidine), poly(acrylates), poly(ethylenesuccinate), poly(vinylalcohol), poly(ethyleneimine), poly(alkylenesulphides) etc. [13].

Improvement of solid polymer electrolytes conductivity can be achieved by the modification of polymer architecture and by using additives. Flame retardancy in lithium batteries is also a major challenge for battery manufacturers. Especially under abusive conditions, full size batteries may undergo thermal runaway that generates a sharp rise in temperature, with potential for explosion and/or fire. Fire-retardant polymer electrolytes are key materials for safer operation of lithium batteries. Solid polymer electrolytes systems with fire-retardant polymer matrixes have been investigated in only few cases. One critical approach to improve the safety of lithium batteries is to enhance the thermal stability of materials for batteries. Organic phosphorus-based compounds are usually used as flame retarding components in electrolytes for lithium batteries. For example, alkyl phosphates such as trimethyl phosphate and triethyl phosphate; phosphazenes such as hexamethyl phosphazene; compounds with phosphorus substituents were used [14-17]. Novel safe and non-flammable phosphorus-based electrolytes composed of phosphate as a linking agent with poly(ethylene glycol) were synthesized [18-21]. In our earlier papers we have reported the possibility of obtaining phosphorus containing polymers, respectively polyphosphonates and polyphosphates by interfacial polycondensation in liquid-liquid system [22], gas–liquid system [23,24] and inverse phase transfer catalysis [25].

**Scheme 1:**
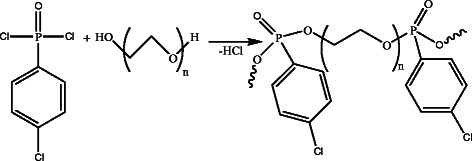
Synthesis of Phosphonate-PEG polymers.

In this paper, novel linear Phosphonate-PEG polymers composed of phosphonate (4-chlorophenyldichlorophosphonate) as a linking agent with poly(ethylene glycol) (PEG) with different molecular weights (MW of 200 and 2000) as soft segment increasing chain flexibility) were synthesized to increase segmental motion to aid the ion transport and ionic conductivity at ambient temperatures (Scheme 1).

In order to improve the parameters of process, increase the yield and reduce the corrosion during the synthesis of the Phosphonate-PEG polymers for SPE, 1-methylimidazole as an acid scavenger was used.

Recently, efforts have been made to perform reactions in greener or more environmentally acceptable media. For providing a minimum of mixing and heat transfer of the exothermal reaction, 1,3-dioxolane was added as green solvent in this synthesis [26].

Triethylamine (TEA) was usually used as scavenger of HCl [27]. Scavenging with a tertiary amine, a thick, non-stirrable slurry results, and low yields were obtained.

Recently, BASF developed an elegant process called BASIL (Biphasic Acid Scavenging using Ionic Liquids) using 1-methylimidazole as HCl scavenger [28,29]. *N*-Methyl imidazole was used as a catalyst in the synthesis of aromatic polyphosphonates [30,31].

The polymers were characterized by IR, ^1^H and ^31^P-NMR, and molecular weights were determined by gel permeation chromatography. The properties of the polymers such as thermal stability and flammability have also been investigated. The phosphonate-PEG polymers which were obtained as solid materials were complexed with lithium triflate and investigated if these copolymers can show potentially applications for electrochemical device. Ionic conductivity and transference number, flammability and elasticity tests for membranes were investigated.

## Results and discussion

Phosphonate-PEG polymers were synthesized by the reaction of 4-chlorophenyldichlorophosphonate and PEG (MW of 200 and 2000) in the presence of TEA or 1-methylimidazole, as HCl scavenger (Scheme 1). HCl is formed during the synthesis of polymers. Hydrochloric acid has to be removed to prevent decomposition of the polymer product. Normally, tertiary amines such as triethylamine are used to scavenge the acids, but these bases form solid salts when they react with acids, and the reaction mixture becomes a suspension. They usually make the reaction mixture more viscous, leading to insufficient mixing of the reagents. These salts can be removed by using an aqueous extraction phase. The slurry causes poor mixing, which leads to poor heat transfer, i.e., hot spots, and difficulty controlling the reaction. The mentioned problems significantly lower the yield and capacity of the process. In order to provide a minimum of mixing and heat transfer of the exothermal reaction usually a solvent has to be added (1,3-dioxolane). 1,3- dioxolane has successfully green applications due to its strong solvency, low odor, superior toxicological profile, and improved safety in handling.

**Scheme 2:**
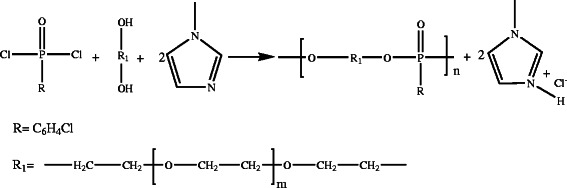
1-Methylimidazole as acid scavenger.

Using 1-methylimidazole as acid scavenger, an ionic liquid is formed:1-methyl-imidazolium chloride, which has a melting point of about 75°C (Scheme 2).

The products can be easily recovered by a simple layer separation, where the upper phase mainly contains the polymer product and the lower layer mostly consists of 1-methylimidazolium chloride (ionic liquid), which could be converted to 1-methylimidazole by treating with NaOH aqueous solution at a room temperature.

Table 1 shows the effect of acid acceptor on the yield and inherent viscosity of the phosphonate-PEG polymers P1-P4.

**Table 1:** Effect of acid acceptor on the preparation of polyphosphonates P1-P4

**Polymer**^**a**^	**Acid acceptor**	**Yield, %**	**η**_**inh**_^**b**^**, dl/g**
P1	TEA	54	0.18
P2	TEA	65	0.35
P3	MeI	75	0.24
P4	MeI	88	0.48

^a.^P1-P4: see Procedure.

^b^measured at a concentration of 0.5 g/dl in tetrachloroethane, at 30°C.

The inherent viscosities of all polymers were in the range 0.18-0.48 dl/ g. The yield of the process decreases in the case of TEA and results indicate that the polycondensation system requires 1-methylimidazole as acid scavenger instead of TEA.

**Figure 1:**
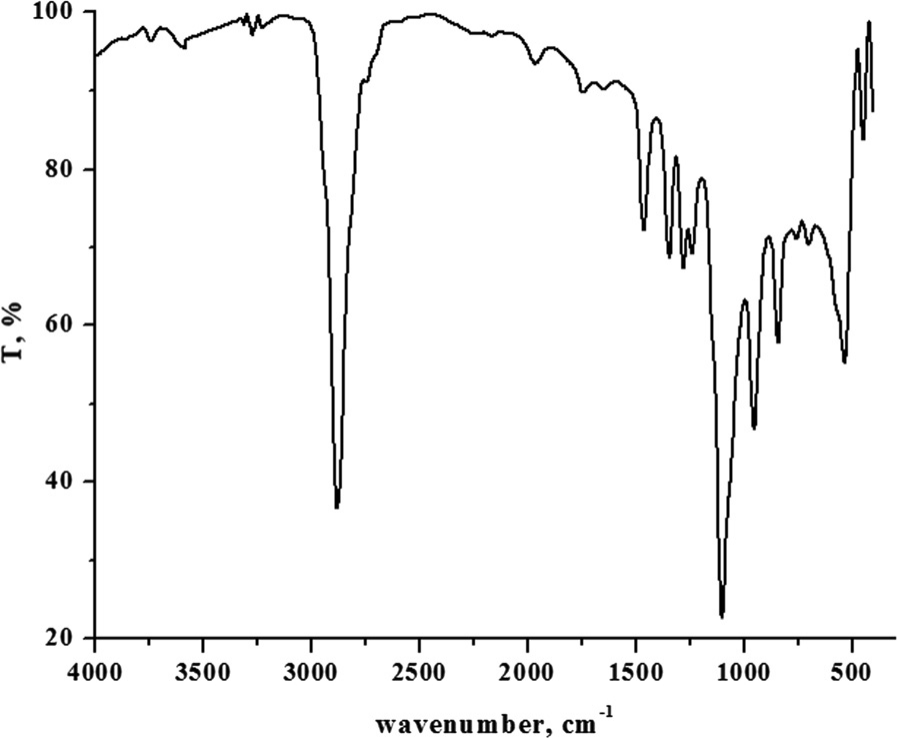
IR spectrum of polyphosphonate P4.

**Figure 2:**
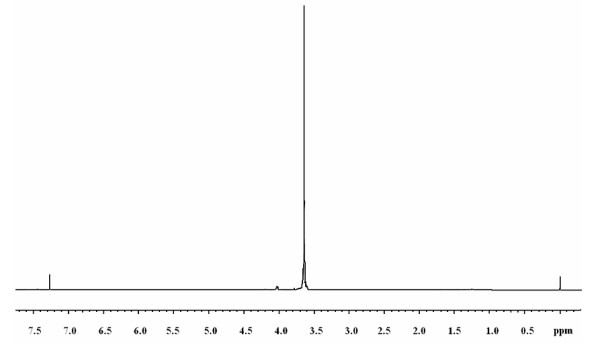
^**1**^**H-NMR spectrum of polyphosphonate P4.**

**Figure 3:**
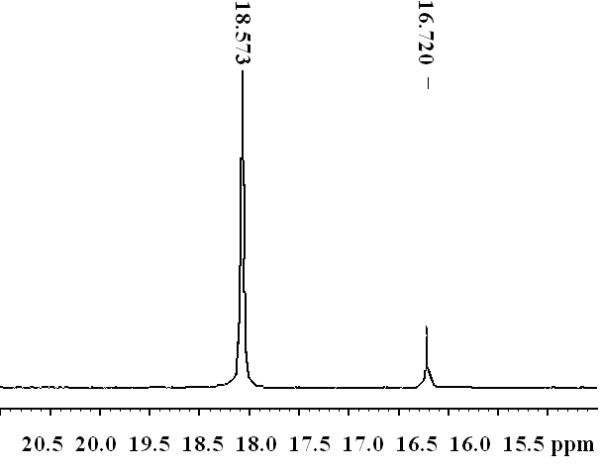
^**31**^**P-NMR spectrum of phosphonate P4.**

**Figure 4:**
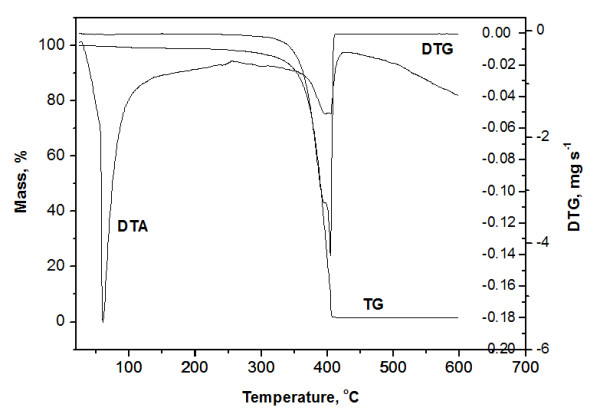
Thermoanalytical curves for polyphosphonate P4.

**Figure 5:**
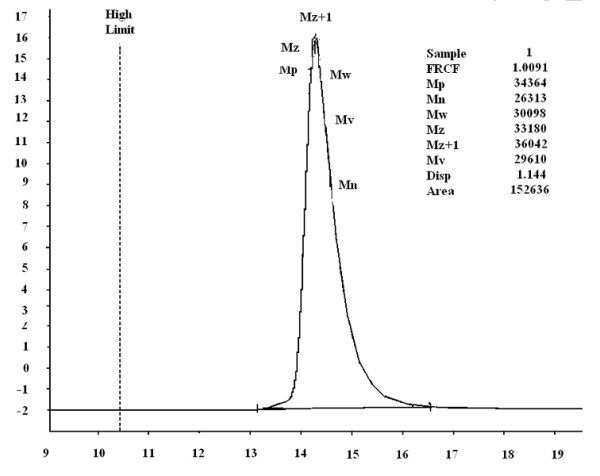
GPC trace of the polyphosphonate P4.

The chemical structure of polymers was authenticated by %P, IR and ^1^H-NMR analysis. IR, ^1^H-NMR, ^31^P-NMR, Molecular weight and distribution, thermogravimetric data for polymer P4 are shown in Figures 1, 2, 3, 4 and 5.

Polymeric structure was supported by P-O-C_aliph_ peaks at 1090–1120 cm^-1^ and 840 cm^-1^, respectively. The disappearance of the phosphonate (P-OH) stretching band at 3500–3200 cm^-1^ indicates that the polymer is indeed a polyphosphonate. Absorptions arising from aliphatic C-H in PEG and P=O stretching of PPD (or CPPD) occur in the region of 2800 cm^-1^ and 1240 cm^-1^, respectively. The resonance of the phenyl group falls in the range 7.0-7.9 ppm [32,33].Chemical shifts of the -CH_2_-O-CH_2_- group were observed at the positions of 3.5-3.7 ppm [34,35].

The presence of phosphorus is confirmed by elemental analysis and ^31^P-NMR spectrum. The %P content of theses polymers is in agreement with the calculated values. The ^31^P-NMR spectra of all polymers present two signals: one corresponds to the P in the repeat unit and other one to the P at the end chain, and confirm successful incorporation of phosphorus in the polymer backbone [36,37].

The thermal stability of the polymers P1-P4 was evaluated by the thermogravimetric analysis and flame retardancy by Limiting Oxigen Index (LOI) (Table 2).

**Table 2:** **Characterization of polymers P**_**1**_**-P**_**4**_

**No**	**P,**^**a**^**%**		**Mn x10**^**-4**^**g/mol**	**Mw x10**^**-4**^**g/mol**	**Poly Disp.**	**Temperature (°C) corresponding to weight loss: 5% 95%**	**T**_**m,**_**°C**	**Residue at 500°C, %**	**LOI, %**
	**calc**	**exp**							
P1	8.29	7.25	0.46	0.75	1.63	328.00 403.00	54.20	1.278	27
P2	1.42	1.12	2.24	2.80	1.25	345.50 410.00	68.20	1.398	26
P3	8.29	7.50	0.51	0.57	1.13	330.12 405.00	55.61	1.321	29
P4	1.42	1.28	2.63	3.00	1.14	352.50 412.00	69.51	1.403	28

^a^determined by Schöniger method.

From the TG data of PEG 2000, a rapid mass loss process beginning at about 345°C and completed at about 420°C can be noticed. This process corresponds to the cleavage of PEG backbone.

For polymers obtained from PEG 200, the rapid mass loss process begins at 328°C and is completed at about 403°C for P1 and for P3 begins at 330.12°C and is completed at 405°C. For polymers obtained from PEG 2000, P2 and P4, the rapid mass loss process begins at 345.5°C and 352.50°C and is completed at about 410°C and 412°C. The mass loss was about 95% for all samples untill 420°C. The residual mass at 500°C was for all samples higher than for PEG 2000 (1.181%). The decrease of rapid mass loss process comparatively with PEG and an increase of residue at 500°C is an indication of polymer formation. The temperature corresponding to 5% weight loss indicates the temperature until polymer is considered to be stable. These temperatures are higher in the case of polymers P2 and P4 than for P1 and respectively P3. An increase of (EG) units in polyphosphonate improves polymer thermal stability. A melting process was observed on DSC curves for all samples. The melting temperature (Tm) values for the synthesized polymers determined from DSC are higher than the PEG used (−38°C for PEG-200 and 49.52°C for PEG-2000, values available from Merck product data sheet).

The molecular weights and polydispersity for the polymers P1-P4 are presented in Table 2. Higher molecular weights were obtained for polymers synthesized by polycondensation of CPPD with PEG 2000 (Figure 5). Also, it was noticed that using TEA as acid scavenger the polymers are obtained with an increase polydispersity, most likely due to the low heat transfer during polycondensation.

Synthesized compounds were tested as flame retardants using LOI method. These polymers show LOI values in the range 26–29, comparable with other polyphosphonates and polyphosphates [38].

Based on the polymer P3 and P4 obtained by method b with MeI as acid scavenger, different membranes with variable lithium salts contents were prepared. The membranes with higher salt concentration (20%) are opaque, smoother but brittle on the margins. The membranes with lower salt concentration (5%) are transparent and harder. The membranes with middle salt concentration (10-15%) are semi-transparent and softer. These interesting tendencies in physical properties of the membranes are the consequence of the decrease in flexibility of chains due to an increase in intramolecular and intermolecular coordination between active sites of the polymer chains. The complexation of polymers with lithium salts allowed the ions to act as transitory cross links [39]. Also, the surface of the membrane containing high salt concentration is less smooth compared with the low and middle salt concentration membranes.

The thermal stability of the membranes based on the polymers P3 and P4, flame retardancy, conductivity and ion transference number were investigated. The obtained results are presented in Table 3.

**Table 3:** Characterization of solid polymer electrolytes membranes based on P3 and P4

**Membrane**	**Tm**_**,**_**°C**	**LOI, %**	**Conductivity at 25°C, S.cm**^**-1**^	**T ion**
P3-5/Li	55.12	28	6.08 × 10^-8^	0.92
P3-10/Li	54.60	28	9.05 × 10^-8^	0.89
P3-15/Li	53.12	29	1.34 × 10^-7^	0.87
P3-20/Li	51.50	28	3.09 × 10^-8^	0.88
P4-5/Li	67.80	29	1.17 × 10^-7^	0.96
P4-10/Li	65.20	29	6.27 × 10^-7^	0.96
P4-15/Li	63.00	28	5.07 × 10^-7^	0.94
P4-20/Li	62.18	28	2.5 × 10^-7^	0.92

The complexation of polymers with lithium salt affects the melting temperature, Tm, of the membrane. Comparatively with parent polymers P3 and P4, lower lithium salt concentration in membranes induces a decrease of Tm with ~1-2°C and higher salt concentration induces a decrease with ~3°C- 7°C. This behavior is attributed to the process of the inhibition of crystallization by the presence of the salt (practically a reduction in crystalline fraction). Even at lower salt concentration a decrease of Tm takes place due to the intercalation of lithium ions and decrease in polymer–polymer interaction.

The LOI values for membrane lies in the range closely to the parent polymers P3 and P4. The presence of the phosphonate group reduces flammability both of the parent polymers and membranes based on them.

**Figure 6:**
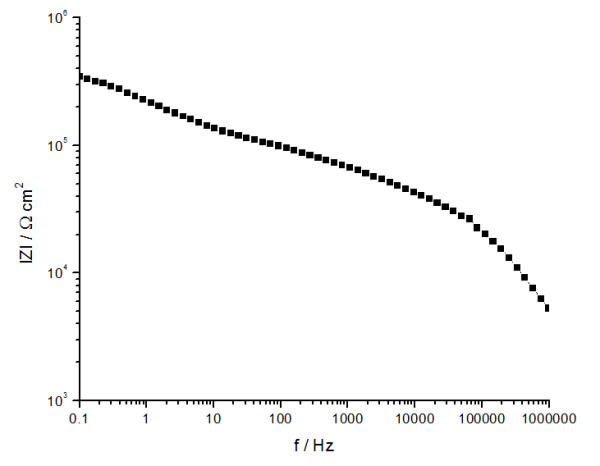
Bode plots at OCP potential, for the studied membrane P4-10/Li.

**Figure 7:**
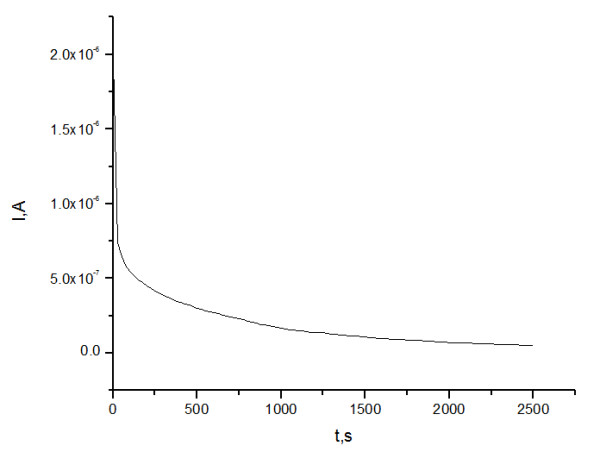
Polarization current as a function of time for membrane P4-10/Li.

In order to evaluate if the polymers P3 and P4 can be used as polymer electrolyte for SPE, the ionic conductivity at different temperatures and ion transference number were determined. The ionic conductivity (σ) value was calculated at room temperature according to Eq. (2), from the intercept of the curve with real axis and the total ionic transference number was calculated from plots of the polarization current versus time with the Eq. (3). The Bode plots for P4-10/Li at Open Circuit Potential (OCP), V, and the evolution of polarization current as a function of time, after the application of a direct current (DC) potential (1.5 V) across the SS/P4-10/Li /SS cell is presented in Figures 6 and 7.

The difference between the membranes lies in the structure of polymer (P3 or P4) and in the salt content. The data indicate that the polymer with a greater amount of EG unit (P4) facilitates the ion transport showing higher ionic conductivity. At the same salt content, the highest conductivity was obtained for membrane based on P4. Also, the conductivity increases with the lithium salt content from 5 to 10% at room temperature and decreases with the increase of lithium salt content from 10 to 20%. The decrease of conductivity was attributed to the decrease of the available number of charge carriers due to ion aggregation. The P4-10/Li membrane presents the highest conductivity of 6.27 × 10^-7^ S.cm^-1^. This value is higher than the conductivity observed for pure PEG 2000, of 1.67 × 10^–9^ S.cm^-1^ and close to 7.27 × 10^–7^ S.cm^-1^ for (PEG)*x*LiClO_4_ system [12].

The conductivity of all membranes increases with the increase of the temperature. This tendency leads to the increase of ions and chains mobility. The polymer can expand easily when the temperature increases, the free volume increase and polymer segments can be without difficulty in motion. Practically, these enhance in mobility can compensate the retarding effect of the ion clouds.

**Figure 8:**
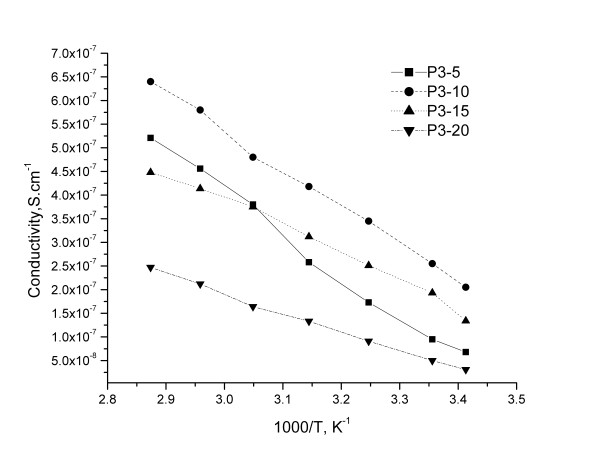
The evolution of conductivity as a function of the temperature for membranes based on P3.

**Figure 9:**
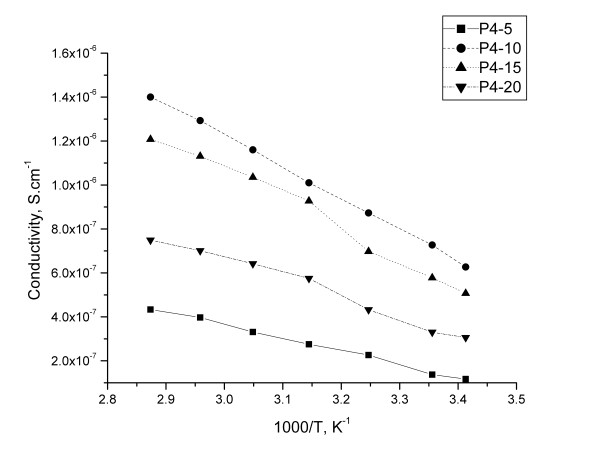
The evolution of conductivity as a function of the temperature for membranes based on P4.

The variation of the conductivity vs. temperature islinear, which is characteristic for a thermally activated process (Figure 8 and 9). It was observed only a slightly modification of the slope around Tm for P3-5/Li and P4-15/Li, due the melting of the crystalline phase, which usually improves the dynamic properties of the polymer electrolytes with an enhancement in the ion conduction [19].

The activation energy Ea values were calculated from the linear segment of all plots using the equation (1):

(1) $$\sigma \left(T\right)=\sigma_{0}\exp\left(-\frac{E_{a}}{kT}\right)$$

Were σ_0_ represents the pre-exponential factor in S.cm^-1^; Ea is the activation energy in eV,; k is the Boltzmann constant.

The activation energy, Ea can be calculated from the slope and the pre-exponential factor can be obtained from the intercept with the vertical axis.

**Figure 10:**
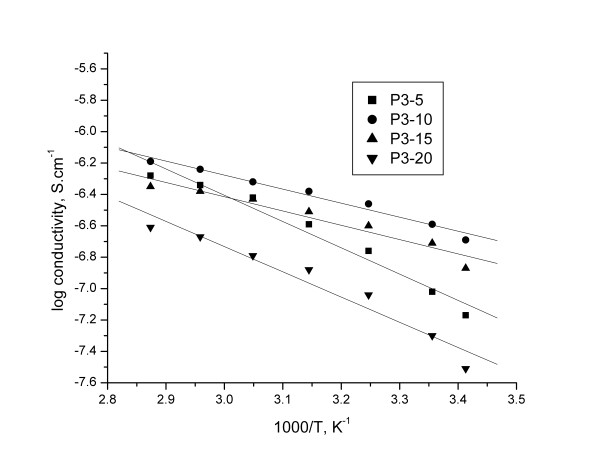
The conductivity plotted in Arrhenius coordinates for membranes based on P3.

**Figure 11:**
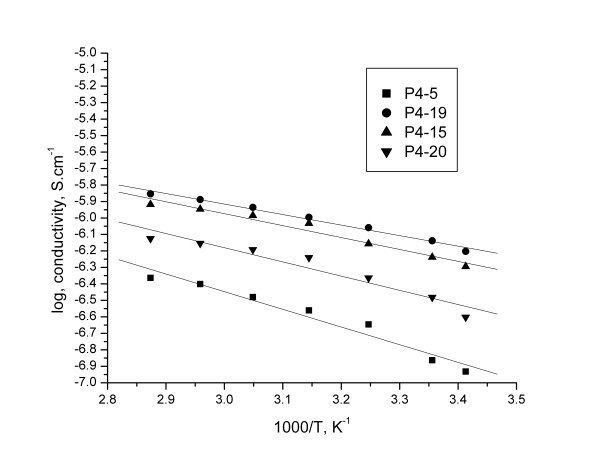
The conductivity plotted in Arrhenius coordinates for membranes based on P4.

The conductivity plotted in Arrhenius coordinates is presented in Figure 10 and 11 (log conductivity vs. 1000/T), all samples fits well the Arrhenius equation.

The pre-exponential factor and activation energy Ea calculated from the linear segment of all membranes are presented in Table 4.

**Table 4:** The pre-exponential factor and activation energy Ea

**Lihium salt, % wt**	**σ**_**0**_**, S. cm**^**-1**^		**E**_**a**_**, eV**		**R**	
	**P3**	**P4**	**P3**	**P4**	**P3**	**P4**
5	1.38266	3.23101	1.67421	1.07206	0.98262	0.97916
10	3.59694	3.9991	0.8934	0.63855	0.98808	0.99301
15	3.68579	3.80389	0.90964	0.72339	0.96845	0.98286
20	1.91187	3.59556	1.60686	0.86164	0.97328	0.96526

The regression values R are close to unity and, therefore, the dependence of ionic conductivity on temperature, for all the complexes, obeys Arrhenius law (R^2^ lies between 0.965 and 0.993) indicating a thermally assisted conductivity mechanism.

The conductivity of polymer electrolytes is affected by the number of charge carriers and their mobility [40]. The low activation energy values were obtained for membranes with higher amount of EG unit (P4). The low activation energy value obtained for P4 membranes indicates that the ion transport is possible by the lower energy barrier to the ions transport in this copolymer matrix.

The structure of the P4 polymer is capable to reduce the apparent activation energy for ion transport and also to increase the local free volume of the matrix. The activation energy depends on salt content which is the source of charge carriers in the polymer electrolyte (the decrease activation energy is due to the increase number of charge carriers in the polymer electrolyte). It was observed a decrease of activation energy with the increase of salt content till 10% and at higher salt concentration an increase of activation energy. This is a consequence of two factors: the first is the number of charge carriers and second is the tendency to aggregate. Practically, the increase in number of charge carriers with the increase of salt content can be overcome by the effect of the ion clouds.

At high salt concentration in membrane the salt exists as ion pairs or aggregated ions that could slow down ion transport resulting in a decrease of conductivity.

The σ_0_ values are highest for P4 membranes and lowest for all P3 membranes. The highest values explain the increase of conductivity in this polymer as a result of the higher quantity of mobile charge carriers in the membranes. A greater amount of EG unit in the P4 matrix shows an improvement of polymer complexation.

The addition of an optimum content of salt 10% provides the most suitable environment for ion transport and achieves the highest conductivity. Similar observations are also reported by Jeon et al. [41].

The total ionic transference number was found to be in the range of 0.94–0.96 and suggests that the charge transport is predominantly due to ions. Also, the total ionic transference number is higher for membranes based on P4 and confirms the observation regarding the improvement of salt complexation due to the greater amount of EG unit in the P4.

**Figure 12:**
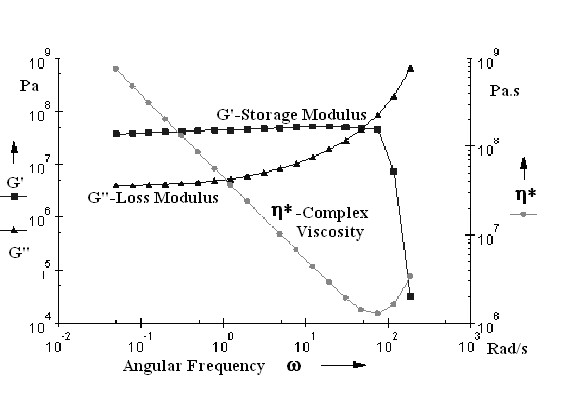
Dynamic mechanical analysis of polymeric membrane based on P4-10/Li; G', G" and η versus angular frequency ω.

**Figure 13:**
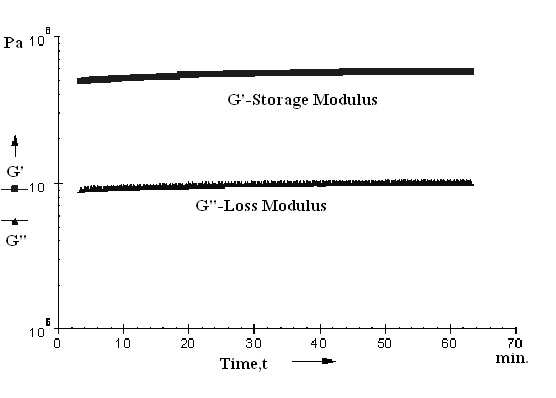
Dynamic mechanical analysis of polymeric membrane based on P4-10/Li; G', G" versus time.

The elasticity tests were performed by measurement the shear stress, and the elasticity modulus. The shear stress and the elasticity modulus were plotted versus angular frequency and time, respectively (Figures 12 and 13).

The membrane based on P4-10/Li showed elasticity modulus of G’ = 5.74 × 10^7^ Pa, the breaking appeared at frequency of ω = 75.95 rad/s.

Maximum conductivity at room temperature with good mechanical stability and total ionic transference number has been observed for P4-10/Li membrane from the various compositions studied. Higher conductivity and good thermal stability of this membrane could be attributed to the optimum composition, with a good balance between EG unit and salt content.

## Experimental

### Materials

Reagents (4-chlorophenyldichlorophosphonate (CPPD) and poly(ethylene glycol) (200 and 2000) from Aldrich, were used as received. 1-methylimidazole (MeI, Aldrich), triethylamine (TEA, Aldrich), solvent (1,3-dioxolane Aldrich) were used without purification. Lithium trifluoromethanesulfonate (lithium triflate) from Aldrich was used after drying under reduced pressure at 120°C for 24h. Methanol (without purification, Aldrich) and ethylene carbonate (EC, without purification, Aldrich) were used for membrane formation.

### Procedure

#### Synthesis of Phosphonate-PEG polymers, P1-P4, by solution polycondensation

Polyphosphonates were synthesized using two routes:

a. triethylamine (TEA) as an HCl scavenger (P1=CPPD+PEG200+TEA; P2=CPPD +PEG2000 +TEA)

b. 1-methylimidazole as an HCl scavenger (P3=CPPD+PEG200 +MeI; P4=CPPD +PEG2000 + MeI)

a. A solution of CPPD (1mmol) in 1,3-dioxolane (5 mL) was added dropwise over 1h to a stirring mixture of PEG (200 or 2000) (1mmol), triethylamine (2 mmol) and 1,3-dioxolane (5 mL) at 25°C.

The reaction mixture was stirred 6 h at 30°C under continuous stirring. After the reaction was completed, HCl^.^TEA side product was filtered 4–5 times. The 1,3-dioxolane was removed by the vacuum evaporation on a rotary evaporator. The products (P1) and (P2) were white solids. Polymers were dried under vacuum, at 50°C.

P1 *IR* (ATR, cm^-1^): 1241.30 (P=O), 1090.36; 844.52 (P-O-C_aliph_), 1461.24 (P-C_arom_); 2879.34 (aliph-CH_2_-); 3048.3;1343.18;1620.91; 755.95 (Ph); 1743.33 (P_arom_-Cl)

^*1*^*H-NMR* (400 MHz, CDCl_3_, TMS) 7.2-7.8 (m,C_6_H_5_); 3.5-3.6 (m, -CH_2_-O-CH_2_-)

P2: *IR* (KBr, cm^-1^): 1240.80 (P=O), 1099.26;840.52 (P-O-C_aliph_), 1463.71(P-C_arom_); 2878.24(aliph-CH_2_-); 3048.3;1343.18; 1620.91;755.95 (Ph); 1742.25 (P_arom_-Cl)

^*1*^*H-NMR* (400 MHz, CDCl_3_, TMS) 7.0-7.6 (m,C_6_H_5_); 3.4-3.7 (m, -CH_2_-O-CH_2_-)

^*31*^*P-NMR* (400 MHz, CDCl_3_, TMS): 12.0(P at chain end) and 15.7 (P in the repeat unit)

b. A solution of CPPD (1 mmol) in 1,3-dioxolane (5mL) was added dropwise over 1 h to a stirring mixture of PEG (200 or 2000) (1 mmol), 1-methylimidazole (2 mmol) and 1,3-dioxolane (5 mL) at 25°C.

The reaction mixture was stirred for 3h at 30°C, then the mixture was heated at 75°C. After the reaction is completed two clear liquid phases occurred that can be easily separated. The upper phase is the polymer solution and the lower phase is the pure ionic liquid. The ionic liquid formed in the lower layer was separated, and then washed with sodium hydroxide to recycle the 1-methylimidazole. The 1,3-dioxolane solution was removed by vacuum evaporation on a rotary evaporator. The products P3 and P4 were white solids. Polymers were dried under vacuum, at 50°C.

P3: *IR* (ATR, cm^-1^): 1242.61 (P=O), 1120.29;842.51 (P-O-C_aliph_), 1460.25 (P-C_arom_); 2878.24(aliph-CH_2_-)3048.3;1343.18; 1620.91; 755.95 (Ph); 1746.23 (P_arom_-Cl)

^*1*^*H-NMR* (400 MHz, CDCl_3_, TMS) 7.2-7.8 (m,C_6_H_5_); 3.5-3.7 (m, -CH_2_-O-CH_2_-)

P4: *IR* (KBr, cm^-1^): 1239.04 (P=O), 1100.19;841.77 (P-O-C_aliph_), 1462.74 (P-C_arom_); 2878.24(aliph-CH_2_-)3048.3;1343.18; 1620.91; 755.95 (Ph); 1743.33 (P_arom_-Cl)

^*1*^*H-NMR* (400 MHz, CDCl_3_, TMS) 7.3-7.9 (m,C_6_H_5_); 3.72 (s,-CH_2_-O-CH_2_-)

^*31*^*P-NMR* (400 MHz, CDCl_3_, TMS): 16.7(P at chain end) and 18.6 (P in the repeat unit)

#### Preparation of solid polymer electrolytes

Polymer electrolytes membranes were prepared based on P3 and P4 polymers obtained with MeI as acid scavenger. Certain amount of lithium salts (i. e. lithium triflate), was added to the melted polymer and stirred until lithium salts is dissolved. Ethylene carbonate (2%) as a plasticizer was added to a phosphonate–polyether (P3 or P4) network to improve ionic conductivity at room temperature. Then the mixture was cast on a Teflon plate and dried in vacuum at 70°C for 24 h to form polymer electrolyte complex films. The membrane notation is P3-m/Li or P4-m/Li, were m represents the weight in % of lithium salt. The notation and composition of prepared lithium polymers membranes were presented in Table 5.

**Table 5:** The notation and composition of prepared membranes

**Membrane notation**	**Polymer**	**Litium salt m, %**	**EC, %**
P3-5/Li	P3	5	2
P3-10/Li	P3	10	
P3-15/Li	P3	15	
P3-20/Li	P3	20	
P4-5/Li	P4	5	
P4-10/Li	P4	10	
P4-15/Li	P4	15	
P4-20/Li	P4	20	

### Analysis

The IR spectra were recorded on a JASCO - FT/IR-4200 spectrophotometer and ^1^H-NMR and ^31^P-NMR spectra on a Bruker DRX 400 MHz spectrometer. All NMR spectra were recorded in CDCl_3_ using TMS as internal standard, at 25°C. The polymer was characterized by viscosity, on an Ubbelohde suspended level viscometer, at 30°C and by gel permeation chromatography (GPC), on an Evaporative Light Scattering Detector, PL-EMD 950 (2x PL gel MIXEDC 300 × 7.5 mm columns; T=35°C; DMF as solvent; Flow 1 ml/min; calibration with KIT polystyrene as standard). The thermoanalytical curves TG, DTG and DTA (as heat flow) were drawn up by a TGA/SDTA 851-LF 1100-Mettler Toledo device, in nitrogen atmosphere and heating rates of 10 degmin^-1^. Limiting oxygen (LOI) index was determinates by Limiting Oxygen Index Chamber 340AJH0038 for all polymers and membranes based on ASTM D2863–1997. The elasticity tests were performed using Anton Paar Rheometer.

Ionic conductivity of the membranes was determined by the AC impedance spectroscopy. The impedance tests were carried out in the frequency range from 0.1 Hz to 10^6^Hz using an Autolab 302N potentiostat/galvanostat equipped with the FRA2 impedance module. The sinusoidal potential amplitude was 10 mV. All electrochemical measurements were performed at room temperature (ambient condition). For each spectrum 60 points were collected, with a logarithmic distribution of 10 points per decade. The sample films were sandwiched between symmetrical cells containing blocking stainless steel (SS) electrodes. Analysis of the impedance spectra is based on the Bode diagrams. In this case, the overall impedance and the phase shift between applied voltage and answering current signal are both plotted against the frequency. At the point where the phase angle is zero (or close to zero), the impedance is pure ohmic and the resistance of the membrane can directly be determined and used for the ionic conductivity calculation, by the following equation (2):

(2) σ=L/Rb.A

where:

σ - ionic conductivity, S cm^-1^

Rb – the resistance corresponding to the angle closest to zero in the Bode diagram, Ω

L – the thickness of the membrane between the electrodes, cm

A – the cross-sectional contact area of the measured sample with the electrodes, cm^2^.

The study of conductivity vs temperature was performed in the temperature range of 25°C to 75°C.

Transference numbers were evaluated with Wagner’s polarization technique. The cation transference numbers, *t+* in the electrolytes were determined by monitoring the current as a function of time on application of a fixed DC voltage (1.5 V) across the sample sandwiched between two SS electrodes [42]. The total ionic transference number was calculated from plots of the polarization current versus time with the equation (3):

(3) tion=1−IfIi

Where *I*_*i*_ is the initial current and *I*_*f*_ is the final residual current.

## Conclusions

In order to reduce the risk of fire in lithium-ion battery new polymers containing phosphorus groups and polyethers were developed. Polymers composed of phosphonate as a linking agent and poly (ethylene glycol) s were synthesized in order to increase local segmental motion and improve ion transport. Polymer electrolytes were synthesized by solution polycondensation of 4-chlorophenyldichlorophosphonate as a linking agent and PEG with different molecular weights (MW of 200 and 2000). 1-methylimidazole as an acid scavenger and of 1,3-dioxolane as a green solvent were used. The advantages are: an ionic liquid is formed and the products can be easily recovered by a simple layer separation; the enhancing of polymer yield; reducing of the corrosion problem.

Best results (yield 88%, inherent viscosities 0.48 dl/g and Mn 26000) were obtained with 1-methylimidazole as an acid scavenger and PEG 2000. These polymers and membranes based on these polymers showed good LOI values (in the range 26-29%) and indicate an improvement of the safety of lithium batteries. The membrane based on P4-10/Li showed elasticity modulus of G’ = 5.74 × 10^7^ Pa, the breaking appeared at frequency of ω = 75.95 rad/s. The temperature - conductivity dependence of the copolymer exhibits Arrhenius behavior and the activation energy increases by increasing the content of EG unit. The conductivity increases initially with the lithium salt content until 10% and decrease with the increase salt content. This decrease in conductivity at higher salt concentration is due to the ion aggregation. The P4-10/Li membrane presents the higher conductivity about 6.27 × 10^-7^ S cm^-1^ at room temperature. The total ionic transference number is in the range of 0.92–0.96 and the charge transport is predominantly due to ions.

## Competing interests

The authors declare that they have no competing interest.

## Authors’ contributions

SI, GI synthesized the polymers and prepared the manuscript, NP, LM contributed in obtaining and characterization of membranes, AP, GI , SI characterized polymers, LZ helped to draft the manuscript. All authors read and approved the final manuscript.
